# Update on cannabis in human sexuality

**DOI:** 10.1007/s00213-024-06643-4

**Published:** 2024-07-08

**Authors:** Denis Lissitsa, May Hovers, Michal Shamuilova, Tal Ezrapour, Leehe Peled-Avron

**Affiliations:** 1 The Gonda Multidisciplinary Brain Research Center, Bar-Ilan University, 5290002 Ramat Gan, Israel; 2 Department of Psychology, Bar-Ilan University, 5290002 Ramat Gan, Israel

**Keywords:** Cannabis, Sexuality, Dosage, Frequency, Animal studies

## Abstract

**Rationale:**

Sexuality is a central aspect of being human that encompasses many facets. Cannabis, a widely used psychoactive substance, has been associated with various effects on sexuality. The relationship between cannabis and sexuality is complex and multifaceted, involving physiological, psychological, and social factors.

**Objectives:**

This review aims to provide an overview of the current literature on the effects of cannabis on several sexual functions, including sexual desire, arousal, orgasm, and sexual satisfaction. It also discusses the potential mechanisms underlying these effects, as well as the impact of dose and frequency of use.

**Results:**

This review has revealed a complex relationship between cannabis dosage and its influence on sexuality. It appears that the frequency of cannabis use in humans has been associated with the frequency of sexual activities. Individuals who use cannabis more frequently tend to report higher levels of sexual activity. Moreover, there is a notable gender difference in how cannabis affects sexuality. In addition, we found lower doses of cannabis to be linked to heightened sexual desire and enjoyment, whereas higher doses may lead to a decrease in sexual desire and performance.

**Conclusions:**

Overall, the association between cannabis and sexuality is complex and warrants further research to better understand the psychological and neurological mechanisms that underlie the effect of cannabis on these sexuality functions and its implications for sexual health. To advance in this endeavor, a crucial step is establishing a precise measurement of dosage in human studies.

## Cannabis

### General information

Cannabis Sativa L., commonly known as cannabis or marijuana, is a versatile plant that has a rich cultural heritage. Its historical significance in traditional medicinal systems like Ayurveda (Guy et al. 2004) and Chinese medicine (Mechoulam 1986) can be traced back at least 3000 years, highlighting its prolonged use through the ages. Throughout time, cannabis has served various purposes, encompassing medicine, spirituality, and recreation (Rubin 2011). Its therapeutic benefits have garnered widespread acclaim, making it a symbol of peace and love embraced by counterculture movements. Interestingly, the World Health Organization estimates that a staggering 21,000 plant species of cannabis worldwide are utilized for medicinal purposes (Pertwee 2014). Within cannabis, there exist over 400 different compounds, with more than 100 of them belonging to the class of cannabinoids (Ashton 2001; Hanuš et al. 2016). These cannabinoids are responsible for the plant's behavioral and psychotropic effects. Among them, the primary contributor to these effects is Δ9-THC (THC). Additionally, other cannabinoids such as cannabidiol (CBD), cannabichromene (CBC), and cannabigerol (CBG) are present, which offer medicinal benefits without inducing psychoactive effects. Apart from cannabinoids, the cannabis plant also contains various non-cannabinoid constituents derived from different classes of natural products (ElSohly et al. 2017).

Both animal and human studies suggest that the two main constituents of cannabis, THC and CBD have the same molecular formula and weight (C_21_H_30_O_2_; molecular weight 314.5 g/mol) but have quite different acute effects (Martin-Santos et al. 2012; Hanuš et al. 2016). The captivating psychoactive properties of cannabis primarily arise from its most famous cannabinoid, THC, which is present in its flowers and produces a wide range of pharmacological effects in animals and humans (Adams and Martin 1996). On the other hand, CBD and its biological effects have been the subject of many studies suggesting potential therapeutic applications, including its anti-inflammatory actions in various preclinical models (Burstein 2015).

In the United States and Australia, approximately 10% of those who have ever used cannabis become daily users, with an additional 20–30% using the drug on a weekly basis (Hall et al. 1999). Compared to heterosexuals, sexual minorities are more likely to use cannabis and have cannabis use disorders (Dyar 2022). When consumed, cannabis induces euphoria, relaxation, perceptual alterations, time distortion, and enhances ordinary sensory experiences such as taste and hearing which is expressed in heightened enjoyment of food and music. In social settings, it can evoke contagious laughter and increased sociability (Hall and Solowij 1998). Moreover, cannabis can also lead to adverse reactions, including heightened anxiety, panic, paranoia, and psychosis, which are dose-related and more common in inexperienced users, individuals with anxiety disorders, and those who are psychologically vulnerable (Johns 2001).

### Effects on the brain

Cannabis affects the brain in many ways by interacting with specific endogenous cannabinoid receptors, such as CB1 and CB2. CB1 receptors are distributed primarily in brain and peripheral tissues (Onaivi et al. 2006). These two well-characterized cannabinoid receptors are differentially distributed within the brain, with high concentrations found in neocortical, limbic, sensory, and motor areas (Ashton 2001). Upon consumption, cannabis releases various cannabinoids, which are differentially distributed within the brain and interacting with those specific endogenous cannabinoid receptors that mediate the effects of cannabinoids and marijuana use (Devane et al. 1988; Onaivi et al. 2006). The distribution of these receptors closely mirrors that of injected THC; they encompass various regions within the brain, including the cerebral cortex, limbic areas (such as the hippocampus and amygdala), and subcortical areas (Herkenham et al. 1990).

According to Pertwee (2008), THC acts as a partial agonist on both CB1 and CB2 receptors. The effects THC produces seem to be highly influenced by the levels of expression and signaling efficiency of cannabinoid receptors, as well as the continuous release of cannabinoids. In contrast, CBD exhibits potent antagonistic properties against CB1 and CB2 receptor agonists in cells or tissues where CB1 and CB2 receptors are present.

## Sexuality

In 1966, Masters and Johnson coined the term: sexual response cycle. The sexual response cycle includes phases of desire, excitement, plateau, orgasm, and resolution (Kaplan 1979; Masters and Johnson 1966). In this section we will introduce and discuss the stages of desire, excitement, and orgasm.

### Sexual arousal and desire

Human sexual arousal is a complex and multidimensional experience encompassing both physiological and psychological processes. It is initiated by the processing of external stimuli (e.g., visual, tactile) or internal stimuli (e.g., fantasy) (Geer et al. 1993) and consists of interconnected components, including physiological changes, emotional expression, and motivated behavior (Frijda 1986). In men, genital response associated with sexual arousal tends to be more strongly associated with subjective sexual arousal to sexual stimuli compared to women (Chivers et al. 2010).

Sexual desire, also known as libido, is a persistent sexual drive or interest that continues throughout the sexual experience until orgasm or satisfaction is achieved (Kaplan 1979). Cherkasskaya and Rosario (2019) found that sexual desire exists on a spectrum, ranging from absent or diminished to high desire. Without desire, individuals may not experience the excitement phase or subsequent stages of the sexual response cycle, as the mental state plays a significant role beyond physical desire and arousal (Basson 2008).

Both men and women experience physiological responses during the excitement phase of sexual cycle response, including myotonia (increased neuromuscular tension throughout the body) and vasocongestion (swelling of genital tissues due to increased blood flow). Vasocongestion can lead to lubrication in women and erections in men. However, it is important to note that vaginal lubrication alone is not an accurate indicator of arousal, as women can exhibit genital responses without experiencing desire (Chivers and Bailey 2005). The plateau phase follows the excitement phase and involves further increase in sexual arousal while sexual tension levels off yet does not reach the threshold required for orgasm. Orgasm, on the other hand, is the release of accumulated sexual tension, resulting in involuntary rhythmic contractions in genital region. However, it should be noted that orgasm is not solely localized to the pelvic region but is a whole-body response (Kolodny et al. 1979).

### Sexual satisfaction and orgasm

Sexual satisfaction is a multifaceted concept that encompasses both emotional and physical fulfillment (Basson 2001) and refers to an individual's subjective assessment of the positive and negative aspects of their sexual relationships (Lawrance and Byers 1995). It can be influenced by various factors, including the quality of the relationship, physical health, and overall well-being (Pascoal et al. 2018). Research has shown that higher sexual satisfaction is correlated with factors such as experiencing multiple and consistent orgasms and engaging in frequent sexual activity (Kontula 2009; Kontula and Miettinen 2016).

Orgasm, typically resulting from rhythmic stimulation of highly sensory receptor-rich body parts, is commonly associated with sexual satisfaction (Komisaruk and Whipple 2011). “Orgasm inequality” is a phenomenon of men having routine and consistent orgasms, while women do not (Mintz 2017). The existence of an orgasm inequality gap highlights the inconsistency in orgasm experiences, particularly among women. Nonetheless, research consistently indicates the importance of orgasm in overall sexual satisfaction (Kontula and Miettinen 2016; Pascoal et al. 2018). While orgasm is often considered a central aspect of sexual satisfaction (Barrientos and Páez 2006), there is ongoing discourse challenging the emphasis on orgasm as the sole measure of satisfaction. Critics argue that this outcome-driven model overlooks and devalues other diverse sexual experiences (Frith 2013; Holmberg and Blair 2009; Potts 2000).

### Sexual disorders

In DSM-5-TR sexual dysfunction is defined as “a clinically significant disturbance in a person’s ability to respond sexually or to experience sexual pleasure” (American Psychiatric Association 2022). Sexual disorders are an important aspect of sexual experiences. In this paper we focus on several dysfunctions which were researched in the context of their interaction with cannabis: vulvodynia, dyspareunia, female anorgasmia, and erectile dysfunction. Vulvodynia is described as persistent vulvar pain lasting longer than three months where there is no recognizable organic cause of the disease, and with several potential associated factors (Bornstein et al. 2016). It is often described as a burning pain provoked by pressure to the vestibule, such as in vaginal penetration, gynecologic examinations, or tampon insertion (Bergeron et al. 2001). Dyspareunia refers to recurring or persistent pain experienced during sexual intercourse (American Psychiatric Association 2022). Female anorgasmia, another sexual disorder, involves marked delay, infrequency, or absence of orgasm and is recognized as the second most common sexual dysfunction in women (Adam et al. 2015). Erectile dysfunction is a prevalent male sexual disorder characterized by the difficulty in achieving or maintaining an erection sufficient for sexual intercourse (Shamloul and Bella 2011). These sexual dysfunctions can impact sexual satisfaction and overall sexual experiences, highlighting the complex interplay between desire, arousal, pain, and orgasm in individuals' sexual lives.

## General effects of cannabis on sexuality in humans

Cannabis, widely believed to enhance sexual desire, has been found to have a bidirectional effect on sexual functioning. On the one hand, cannabis use has been associated with positive outcomes, such as prolonged intercourse, improved orgasm quality, and increased sexual satisfaction in both men and women; conversely, cannabis use has been linked to negative effects, including erectile dysfunction, sterility, and reduced testosterone levels. Additionally, cannabis use has been correlated with decreased condom use and a higher risk of sexually transmitted diseases (Balon 2017; Bustamante et al. 2022; Scimeca et al. 2017).

Interestingly, over 70% of participants report increased desire and orgasm intensity. Cannabis use has been linked to an increase in masturbation frequency (Barbonetti et al. 2024), with individuals who engage in masturbation reporting heightened pleasure when using cannabis (Moser et al. 2023). Additionally, many individuals noted an improvement in their sense of taste and touch during sexual experiences while under the influence of cannabis (Moser et al. 2023). Furthermore, cannabis has been reported to be used for sex to increase sexual pleasure, lower inhibitions, reduce feelings of anxiety and shame, and foster intimacy and connection with sexual partners (Parent et al. 2021).

Cannabis affects sexual function differently in men and women. According to a study of women with difficulty achieving orgasm, cannabis use before partnered sex eased reaching orgasm, improved orgasm frequency and satisfaction with orgasm (Mulvehill and Tishler 2024). Research suggests that cannabis may benefit women by assisting with conditions like dyspareunia, vulvodynia, pelvic pain, and other urogenital painful conditions. Cannabis may also improve symptoms of painful bladder urgency and enhance orgasms and overall sexual satisfaction. Conversely, in men studies predominantly show that cannabis increases the risk of erectile dysfunction (Lyzwinski 2023).

Nevertheless, due to ethical constraints, no recent studies have utilized objective measurements to explore cannabis' impact on human sexuality, prompting us to investigate objective studies using non-human animals.

## Effects of cannabis on sexuality in non-human animal studies

Research concerning the effects of cannabis on male animals primarily centers on penile erection. Studies indicate that cannabis exerts a positive influence on penile erections in non-human subjects through two distinct mechanisms:

1. Studies by Melis and colleagues have demonstrated that erectile function is modulated by a specific group of oxytocinergic neurons in the paraventricular nucleus of the hypothalamus (PVN) of male mice, which contain CB1 receptors. Inhibiting these receptors has been shown to induce erections (Argiolas and Melis 2005; Castelli et al. 2007; Melis et al. 2004). This mechanism is believed to involve increased glutamatergic signaling, leading to enhanced nitric oxide production by oxytocinergic neurons, resulting in the release of oxytocin that facilitates penile erection (Castelli et al. 2007; Melis et al. 2006).
2. Cannabinoids may also impact penile erection through the smooth muscle in the corpus cavernosum, which is essential for initiating and sustaining erections. Studies have revealed the presence of CB1 receptors in the corpus cavernosum of rats, as well as CB1 and CB2 receptors in the corpus cavernosum of rhesus monkeys and humans (Gratzke et al. 2010; Melis et al. 2006).

Compared to males, the effects of cannabis on sexual behavior in female animals have been less studied and understood. This is attributed to factors such as the cyclic nature of the reproductive cycle, greater behavioral variability, complex hormonal interactions, and the influence of social and environmental factors.

In general, in non-humans the challenges of studies of this type are different – aspects of sexuality are harder to measure directly, and so only aspects that are simple to measure have been studied.

Despite these challenges, animal models provide valuable insights into cannabis' potential objective effects on sexuality.

## Effects of dose and frequency

After an extensive examination of the existing literature, it becomes evident that two key factors, frequency, and dose, have emerged as the focal points in understanding the impact of cannabis on human sexuality.

### Frequency

The frequency of cannabis use plays a crucial role in understanding its effects on sexuality. Studies examining the relationship between cannabis usage frequency and sexual functioning have reported both varying and similar effects across genders.

Several studies have examined the effects of cannabis usage on sexual activities and experiences across genders. For instance, Sun and Eisenberg (2017) conducted a study utilizing data from the National Survey of Family Growth, which involved more than 30,000 participants. The study findings revealed a significant association between the frequency of cannabis use and the frequency of engaging in sexual intercourse. In a study by Halikas et al. (1982), 100 regular cannabis users were interviewed and divided into two groups: frequent users (more than 5 times a week) and less frequent users. Interestingly, no significant difference was observed between the two groups in terms of enhanced sensory experiences, such as touch, smell, or taste, during sexual activity. However, the study did reveal that only frequent users intentionally sought to use cannabis to enhance their sexual experiences. This finding was consistent with Dawley et al.'s (1979) study, which indicated that only the more frequent cannabis users perceived cannabis as an aphrodisiac. Additionally, a report by Nahas and Greenwood (1974) from the Commission on Marijuana suggested that frequent cannabis use was associated with a greater increase in sexual pleasure compared to both non-daily and rare use. Conversely, Wiebe and Just (2019) discovered that excessive cannabis use interfered with sexual pleasure.

Exploring the impact of cannabis on female sexual function, a study conducted by Kasman et al. (2020), divided female customers of a cannabis dispensary into four categories based on their frequency of cannabis usage. They were surveyed using the Female Sexual Function Index (FSFI) questionnaire developed by Rosen et al. (2000), which assesses female sexual function over a four-week period across six individual domains with defined cutoff scores for sexual dysfunction. The study revealed a significant difference in total FSFI scores between the highest and lowest frequency categories, favoring higher usage. Increasing cannabis usage frequency by an additional day was associated with higher total FSFI scores, as well as improvements in desire, arousal, orgasm, and satisfaction domains. Furthermore, as the frequency category increased, the likelihood of reporting sexual dysfunction declined. In another study by Lynn et al. (2019) utilizing the FSFI, it was discovered that frequent users had twice the odds of reporting satisfactory orgasms compared to those with infrequent use. A similar observation by Mulvehill and Tishler (2024) revealed that among women experiencing difficulties achieving orgasm, the frequency of cannabis use before partnered sex correlated positively with orgasm frequency. Additionally in a separate survey study by Smith et al. (2010), there was no observed association between frequency of cannabis use and sexual difficulties in women, such as reaching orgasm, dyspareunia, vaginal dryness, or lack of sexual pleasure.

Regarding male sexual function, it was demonstrated that daily cannabis use was associated with difficulties in achieving orgasm, including both delayed and premature ejaculation (Smith et al. 2010). Additionally, the study found no significant association between the frequency of cannabis use and trouble maintaining an erection. In a study conducted by Bhambhvani et al. (2020), the International Index of Erectile Function (IIEF) instrument (Rosen et al. 1997) was utilized to examine the impact of cannabis usage on male sexual function. The study involved 325 men who were categorized into four frequency categories based on their cannabis use. The findings of the study revealed that men who used cannabis more frequently had higher overall IIEF scores. Furthermore, they performed better in four out of the five functional domains of the IIEF, namely erectile, orgasm, intercourse satisfaction, and overall satisfaction. However, no significant association was found between cannabis usage frequency and the sexual desire domain. However, a smaller study found no significant association between the frequency of cannabis use and IIEF scores (Kumsar et al. 2016). Moreover, the same instrument employed in a study among 30 men with cannabis use disorder revealed a negative correlation between frequency of use and all domains of the IIEF (Asal et al. 2024).

**Fig. 1:**
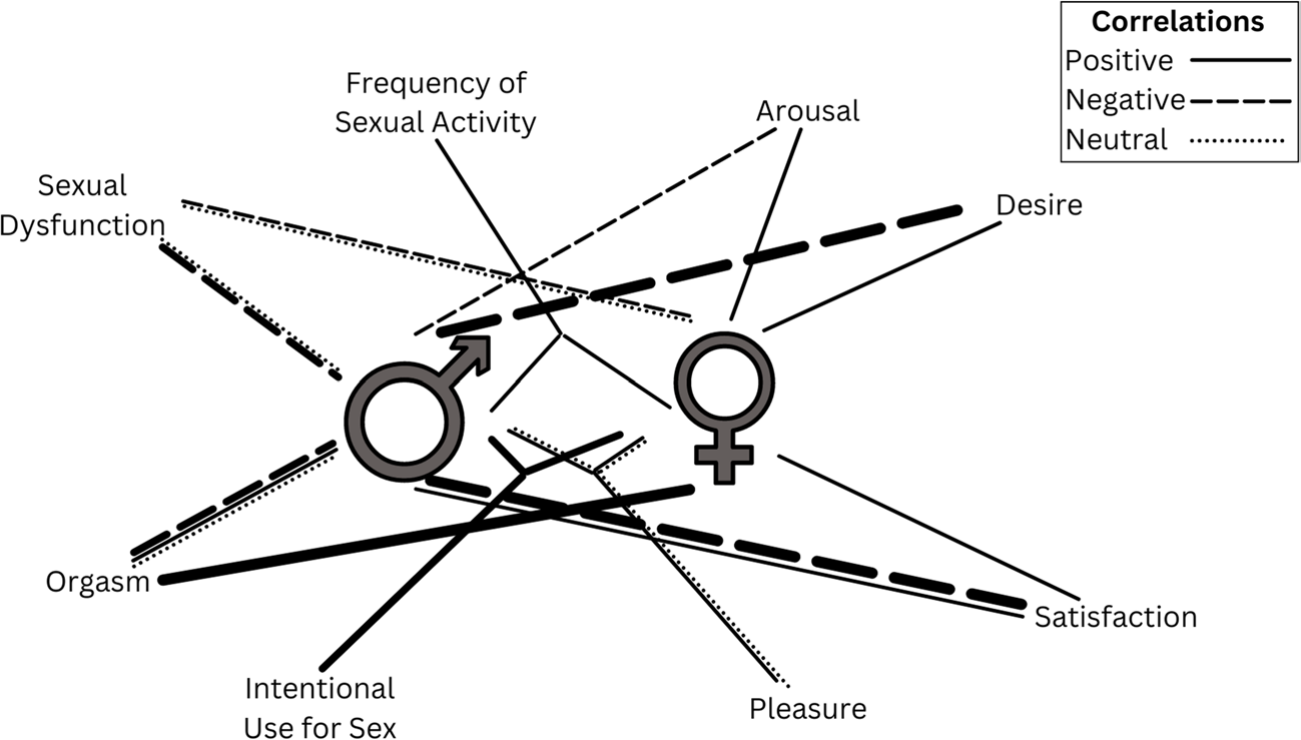
Visual representation of the influence of cannabis use frequency on various aspects of human sexuality, split by gender. The figure illustrates the way heightened frequency is associated with distinct facets of sexuality. Within the visualization, a solid line signifies a study that established a positive correlation with the given aspect, while a dashed line indicates a negative correlation. Additionally, a dotted line portrays a study that detected no discernible effect. Notably, the width of each line corresponds to the number of studies validating the particular finding

In conclusion, studies investigating the impact of cannabis use frequency on human sexuality have yielded diverse and occasionally conflicting findings. Therefore, additional research is necessary, with a focus on standardization of frequency measurement and controlling for more covariates. This will help provide a more comprehensive understanding of the complex interplay between cannabis use frequency and sexuality (Fig. 1).

### Dosage

The dosage of cannabis consumed significantly affects its impact on sexuality, as varying levels of THC and CBD can influence sexual desire, arousal, and satisfaction (Palamar et al. 2018). However, researching the effects of dosage presents challenges due to the difficulty of accurately determining the exact dosage consumed by the average user, as there is no standard cannabis dose.

Despite the challenges surrounding the exploration of cannabis dosage and its impact on sexuality, a few studies have attempted to shed light on this relationship. For instance, in an earlier study conducted by Koff (1974), participants were asked to roll cannabis cigarettes, so that the researchers could estimate the average amount of cannabis in a cigarette. The findings revealed that both men and women who smoked between one and two cannabis cigarettes, with a THC content of 1%, reported an increase in sexual desire and enjoyment. However, beyond that dosage, the positive effects were no longer noticeable. Similarly, it was demonstrated that lower doses of cannabis increased sexual pleasure, while higher doses resulted in a reduction in sexual desire and performance among Indian men (Chopra and Jandu 1976). This association was further substantiated (Abel 1981) emphasizing the correlation between low doses of cannabis and enhanced sexual activity and very high doses were linked to difficulties in sexual performance (Buffum 1982).

In a review of recent studies, Kipping and Lynn (2022) found that moderate doses of cannabis improved female sexual function in domains such as orgasm, libido, and arousal. However, they also noted that high doses of cannabis may have negative effects on female sexual function.

**Fig. 2:**
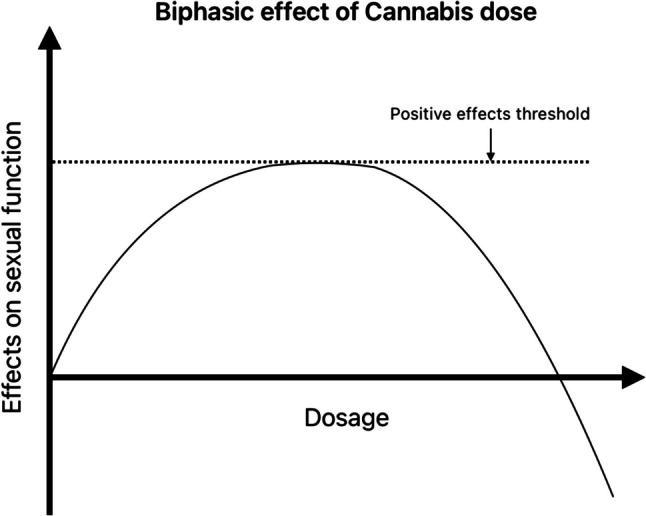
Proposed illustration depicting the biphasic impact of cannabis dosage on human sexual function. This visual representation offers insight into the potential biphasic influence of Cannabis dosage on human sexual function. This conceptual figure is formulated based on our exploration of numerous studies indicating a biphasic trend in Cannabis dosage effects (Chopra and Jandu 1976; Kipping and Lynn 2022). At lower doses, Cannabis appears to exhibit favorable effects on human sexual function (Koff 1974; Abel 1981). These positive effects demonstrate an apparent upper limit, as denoted by the horizontal dotted line. Beyond a specific dosage threshold, the favorable impacts plateau (Koff 1974). Conversely, as dosage increases further, the constructive effects begin to wane, potentially transitioning into adverse effects. This transition is suggestive of a shift from positive to negative influences on human sexual function

These studies collectively highlight the complex inverted U relationship between cannabis dosage and its impact on sexuality, with lower to medium doses generally showing positive effects and higher doses potentially leading to diminished sexual experiences (Fig. 2).

## Discussion

The use of cannabis is prevalent today, with approximately 10% of individuals who have ever used cannabis becoming daily users, and an additional 20–30% using the drug on a weekly basis (Hall et al. 1999). In addition, cannabis use among adults and its availability (as a result of spreading legalization) are on the rise (Hasin and Walsh 2021; Rotermann 2020). Due to these facts, as well as reports of adverse effects of cannabis on humans (Arnold 2021; Hall and Solowij 1998), we decided to investigate how cannabis affects human sexuality.

Our initial investigation led us to question claims made by heavy cannabis users that cannabis acts as an aphrodisiac (Halikas et al. 1982; Koff 1974; Touw 1981), as the users may be biased.

Reports suggest that cannabis has the potential to enhance sexual pleasure, reduce inhibitions, alleviate anxiety and shame, and promote intimacy and connection with sexual partners (Parent et al. 2021). Furthermore, it has been associated with increased pleasure during masturbation and enhanced sensory experiences during sexual encounters (Moser et al. 2023). These observations indicate that cannabis may have notable effects on sexual experiences.

It is important to recognize that sexual satisfaction and the sexual response cycle are complex phenomena influenced by various factors, including the quality of the relationship, physical health, and overall well-being (Pascoal et al. 2018). Cannabis affects individuals in an integrative manner, impacting both physical and emotional aspects, which can potentially influence sexual experiences.

Sexual experiences as well as the effects of cannabis vary between men and women (Matheson et al. 2020; Sholler et al. 2021), those are congruent with studies finding the effects of cannabis on sexual function to differ between women and men. Among the effects on women might being alleviation of conditions such as dyspareunia (painful intercourse) and enhancement of overall sexual satisfaction. Moreover, low doses of cannabinoids, including THC and tetrahydrocannabivarin, which possess sedative and hypnotic properties (Adams and Martin 1996), could potentially alleviate anxiety associated with sexual activities or interpersonal interactions, consequently disinhibiting sexual desire and arousal, particularly in certain women. The results of studies on men are conflicting (Shamloul and Bella 2011)—some suggest that cannabis causes erection dysfunction, premature ejaculation, and postponed ejaculation (Pizzol et al. 2019; Smith et al. 2010), while others claim the opposite (Bhambhvani et al. 2020). Throughout our study, we found the dosage and frequency of cannabis use to be modulating factors in the effects of cannabis on sexual experiences. However, the many conflicting results of different studies raise questions on the validity of the findings.

Most of the studies examined utilized subjective measurements for both sexuality and cannabis use, employing a wide range of questionnaires, contributing to inconsistencies. Thus, our aim was to identify studies employing objective measures. However, ethical constraints primarily confine objective studies to animal models, presenting a limitation as many aspects of sexuality are inherently subjective. Consequently, measurements often rely on observable factors like erections and coupling behavior.

Several factors contribute to the inconsistent findings. Foremost is the variability in ingested dose measurement, as the effects of cannabis derive from the concentration and interaction of its active components, which vary among strains (Ashton 2001; Naim-Feil et al. 2023; Hanuš et al. 2016). Moreover, cannabis consumption methods (e.g., smoking, vaporizing, orally) and preparation techniques influence consumer effects (Farokhnia et al. 2020; Julien 1995). Lastly, within each method of consumption, the concentration of active components differs with the method of preparation. To conclude, cannabis active component variation differs between strains, ingestion method and preparation. Most times, users do not measure the exact concentration of active components in the cannabis they consume, rendering questionnaires that do try to measure dosage unreliable.

Another factor is the notable change in active component concentrations over time. Studies indicate a significant increase in THC concentration and THC/CBD ratio from the 1990s to the mid-2010s, (Dujourdy and Besacier 2017; ElSohly et al. 2016; Freeman et al. 2018) making inferences from older studies undependable.

Furthermore, variability in subjective study nature, including differences in question wording regarding libido, orgasm, pleasure, and pain, as well as diverse assessment methods (e.g., questionnaires, interviews) and focus on specific marijuana users or the general population, contributes to literature inconsistencies.

These limitations, compounded by the absence of double-blind, randomized, placebo-controlled trials involving humans, impede the availability of conclusive evidence.

To mitigate these limitations, we suggest the following measures: Firstly, for questionnaire-based studies aiming to accurately assess dosage, future research should incorporate inquiries about consumption methods with easily measurable active component content. Cannabis edibles serve as a prime example, as legally purchased edibles typically provide such information. Moreover, prioritizing the measurement of dosage is crucial, even if it means excluding participants who cannot provide accurate information, as dosage is fundamental for assessing effects. Additionally, standardizing measurements of sexuality and cannabis usage across questionnaire studies would enhance comparability. Finally, to address the absence of objective human studies and the subjective nature of sexual experience, integrating findings from objective animal studies with those from subjective human studies would help construct a comprehensive understanding of the effects of cannabis on human sexuality.

Overall, the effects of cannabis on human sexuality remain largely elusive and poorly understood. Despite efforts to investigate this complex relationship, our understanding is hindered by several factors, including methodological limitations and challenges in accurately measuring dosage. Future research ought to address these limitations and employ rigorous methodologies to provide more definitive insights into how cannabis influences human sexuality.

## Conclusion

In conclusion, this review paper has examined the extensive body of research exploring the effect of cannabis on sexuality. Throughout the analysis, it has become evident that cannabis exerts a multifaceted influence on various aspects of human sexuality, encompassing both positive and negative outcomes. Animal studies have provided valuable insights into the potential effects of cannabis on sexual behavior in both males and females, but further research, including human clinical trials, is essential to better understand the implications of cannabis use on sexual health and behavior in humans. Moreover, the dose and frequency of cannabis use have emerged as crucial factors in studying its effects on sexuality. Studies investigating the impact of cannabis use frequency on human sexuality have yielded diverse and occasionally conflicting findings, necessitating additional research with a focus on controlling for more covariates. Additionally, the relationship between cannabis dosage and its impact on sexuality appears to be complex and biphasic, with lower doses generally showing positive effects and higher doses potentially leading to diminished sexual experiences. However, these findings are cast into doubt due to the unreliability of dosage measurement in most studies.

Overall, numerous obstacles hinder the study of cannabis effects on human sexuality, and our knowledge is still very limited. Yet a deeper understanding can aid in mitigating harm and potentially enhancing human experiences.
